# Supplementary material for: Metabolic fingerprinting on retinal pigment epithelium thickness for individualized risk stratification of type 2 diabetes mellitus
Source: Nat Commun. 2023 Oct 18;14:6573. doi: 10.1038/s41467-023-42404-1 (PMC10585002; doi:10.1038/s41467-023-42404-1)
Supplement: Supplementary file 1 — Supplementary Information [file 41467_2023_42404_MOESM1_ESM.pdf]

## SUPPLEMENTARY INFORMATION


**SUPPLEMENTARY TABLES** ........ 3

Supplementary Table S1 ........ 3

Supplementary Table S2 ........ 6

Supplementary Table S3 ........ 7

Supplementary Table S4 ........ 8

Supplementary Table S5 ........ 10

Supplementary Table S6 ........ 13

Supplementary Table S7 ........ 15

Supplementary Table S8 ........ 16

Supplementary Table S9 ........ 19

Supplementary Table S10 ........ 21

Supplementary Table S11 ........ 22

Supplementary Table S12 ........ 24

Supplementary Table S13 ........ 26

Supplementary Table S14 ........ 29

Supplementary Table S15 ........ 31

**SUPPLEMENTARY FIGURES** ........ 34

Supplementary Figure S1 ........ 34

Supplementary Figure S2 ........ 35

Supplementary Figure S3 ........ 36

**SUPPLEMENTARY REFERENCES** ........ 37

**SUPPLEMENTARY TABLES**

**Supplementary Table S1**

Baseline characteristics of the included UKB population.

| **Characteristic *** | **Overall** | **Population-I** | **Population-II** | | **P value †§** | **P value ‡§** |
|---|---|---|---|---|---|---|
| | | | **Training set** | **Testing set** | | |
| No. of subjects | 92048 (100) | 7824 (100) | 58835 (100) | 25389 (100) | - | - |
| Age at recruitment | | | | | | |
| *≤49* | 21214 (23.0) | 2248 (28.7) | 13247 (22.5) | 5719 (22.5) | **<0.001** | 0.725 |
| *50-54* | 13616 (14.8) | 1191 (15.2) | 8614 (14.6) | 3811 (15.0) | | |
| *55-59* | 16563 (18.0) | 1319 (16.9) | 10666 (18.1) | 4578 (18.0) | | |
| *60-64* | 22753 (24.7) | 1800 (23.0) | 14668 (24.9) | 6285 (24.8) | | |
| *≥65* | 17902 (19.4) | 1266 (16.2) | 11640 (19.8) | 4996 (19.7) | | |
| Sex | | | | | | |
| *Female* | 50447 (54.8) | 4122 (52.7) | 32416 (55.1) | 13909 (54.8) | **<0.001** | 0.407 |
| *Male* | 41601 (45.2) | 3702 (47.3) | 26419 (44.9) | 11480 (45.2) | | |
| Race | | | | | | |
| *White* | 87172 (94.7) | 7188 (91.9) | 55885 (95.0) | 24099 (94.9) | **<0.001** | 0.824 |
| *Others* | 4464 (4.8) | 589 (7.5) | 2700 (4.6) | 1175 (4.6) | | |
| *Missing* | 412 (0.4) | 47 (0.6) | 250 (0.4) | 115 (0.5) | | |
| Townsend Deprivation Index | | | | | | |
| *Quartile 1* | 23293 (25.3) | 1749 (22.4) | 15056 (25.6) | 6488 (25.6) | **<0.001** | 0.739 |
| *Quartile 2* | 23027 (25.0) | 1861 (23.8) | 14721 (25.0) | 6445 (25.4) | | |
| *Quartile 3* | 22881 (24.9) | 2145 (27.4) | 14551 (24.7) | 6185 (24.4) | | |
| *Quartile 4* | 22724 (24.7) | 2059 (26.3) | 14429 (24.5) | 6236 (24.6) | | |

| | | | | | | |
|---|---|---|---|---|---|---|
| *Missing* | 123 (0.1) | 10 (0.1) | 78 (0.1) | 35 (0.1) | | |
| Education achievement | | | | | | |
| *Level O* | 31643 (34.4) | 2316 (29.6) | 20475 (34.8) | 8852 (34.9) | **<0.001** | 0.823 |
| *Level A* | 4850 (5.3) | 499 (6.4) | 3060 (5.2) | 1291 (5.1) | | |
| *University* | 54509 (59.2) | 5009 (64.0) | 34560 (58.7) | 14940 (58.8) | | |
| *Missing* | 1046 (1.1) | 0 (0.0) | 740 (1.3) | 306 (1.2) | | |
| Average total household income before tax, *£* | | | | | | |
| *< 18k* | 18225 (19.8) | 1214 (15.5) | 11896 (20.2) | 5115 (20.1) | **<0.001** | 0.770 |
| *18k~30k* | 20388 (22.1) | 1627 (20.8) | 13167 (22.4) | 5594 (22.0) | | |
| *31k~51k* | 20320 (22.1) | 1846 (23.6) | 12866 (21.9) | 5608 (22.1) | | |
| *52k~100k* | 15572 (16.9) | 1610 (20.6) | 9699 (16.5) | 4263 (16.8) | | |
| *> 100k* | 3953 (4.3) | 516 (6.6) | 2410 (4.1) | 1027 (4.0) | | |
| *Missing* | 13590 (14.8) | 1011 (12.9) | 8797 (15.0) | 3782 (14.9) | | |
| Body mass index, $kg/m^2$ | | | | | | |
| *Normal* | 29879 (32.5) | 2650 (33.9) | 19077 (32.4) | 8152 (32.1) | **0.044** | 0.463 |
| *Overweight* | 39247 (42.6) | 3311 (42.3) | 25000 (42.5) | 10936 (43.1) | | |
| *Obesity* | 22568 (24.5) | 1839 (23.5) | 14524 (24.7) | 6205 (24.4) | | |
| *Missing* | 354 (0.4) | 24 (0.3) | 234 (0.4) | 96 (0.4) | | |
| Smoking | | | | | | |
| *Never* | 32193 (35.0) | 2711 (34.6) | 20656 (35.1) | 8826 (34.8) | **0.033** | 0.497 |
| *Ever/Current* | 9901 (10.8) | 771 (9.9) | 6395 (10.9) | 2735 (10.8) | | |
| *Missing* | 49954 (54.3) | 4342 (55.5) | 31784 (54.0) | 13828 (54.5) | | |
| Drinking | | | | | | |
| *Never* | 3346 (3.6) | 261 (3.3) | 2174 (3.7) | 911 (3.6) | 0.152 | 0.378 |
| *Ever/Current* | 84524 (91.8) | 7235 (92.5) | 54005 (91.8) | 23284 (91.7) | | |
| *Missing* | 4178 (4.5) | 328 (4.2) | 2656 (4.5) | 1194 (4.7) | | |

| | | | | | | |
|---|---|---|---|---|---|---|
| Systemic hypertension | | | | | | |
| *No* | 66012 (71.7) | 5870 (75.0) | 42016 (71.4) | 18126 (71.4) | **<0.001** | 0.959 |
| *Yes* | 26036 (28.3) | 1954 (25.0) | 16819 (28.6) | 7263 (28.6) | | |
| Hyperlipidemia | | | | | | |
| *No* | 49774 (54.1) | 4323 (55.3) | 31760 (54.0) | 13691 (53.9) | 0.090 | 0.886 |
| *Yes* | 42274 (45.9) | 3501 (44.7) | 27075 (46.0) | 11698 (46.1) | | |
| Lipid-lowering medication | | | | | | |
| *No* | 76522 (83.1) | 6525 (83.4) | 48880 (83.1) | 21117 (83.2) | 0.764 | 0.746 |
| *Yes* | 15526 (16.9) | 1299 (16.6) | 9955 (16.9) | 4272 (16.8) | | |
| Blood pressure-lowering medication | | | | | | |
| *No* | 73024 (79.3) | 6424 (82.1) | 46572 (79.2) | 20028 (78.9) | **<0.001** | 0.377 |
| *Yes* | 19024 (20.7) | 1400 (17.9) | 12263 (20.8) | 5361 (21.1) | | |

* Continuous variables were presented as mean (SD), and categorical variables were presented as number (percentage).

† Comparison between population-I and population-II. Student's t-test and chi-square test were used to compare continuous and categorical variables, respectively.

‡ Comparison between training set and testing set. Student's t-test and chi-square test were used to compare continuous and categorical variables, respectively.

§ Bold indicates statistically significant. Two-sided statistical tests were conducted, and no adjustments were made for multiple comparisons.

**Supplementary Table S2**

Multivariable CPH models for evaluating association between RPET and T2DM risk across sexes and genetic risk of AMD.

| Risk of T2DM *† | HR | 95% CI | P ‡ |
|---|---|---|---|
| Overall population | 0.856 | 0.768–0.954 | **0.005** |
| *Women* | 0.828 | 0.698–0.983 | **0.031** |
| *Men* | 0.852 | 0.740–0.982 | **0.027** |

* Adjusted for age, ethnicity, household income, Townsend deprivation index, education, smoking, drinking, body-mass index, use of antihypertensive, and lipid-lowering medication.

† Per-SD change of RPET, measured by SD-OCT using the ETDRS nine-pane grid.

‡ Bold indicates significant. Two-sided statistical tests were conducted, and no adjustments were made for multiple comparisons.

RPET = retinal pigment epithelium thickness; T2DM = type 2 diabetes mellitus; AMD = age-related macular degeneration; HR = hazard ratio; CI = confidence interval; SD = standard deviation; SD-OCT = spectral-domain optical coherence tomography; ETDRS = Early Treatment Diabetic Retinopathy Study.

**Supplementary Table S3**

Multivariable CPH models for evaluating association between RPET across all ETDRS subfields and T2DM risk in participants with low-moderate PRS risk of AMD.

| Risk of T2DM *† | HR | 95% CI | P ‡ |
|---|---|---|---|
| **Total average** | 0.835 | 0.734–0.950 | **0.006** |
| **Outer average** | 0.739 | 0.606–0.901 | **0.003** |
| *Outer superior* | 0.753 | 0.617–0.919 | **0.005** |
| *Outer nasal* | 0.799 | 0.660–0.968 | **0.022** |
| *Outer inferior* | 0.763 | 0.622–0.936 | **0.010** |
| *Outer temporal* | 0.761 | 0.620–0.934 | **0.009** |
| **Inner average** | 0.787 | 0.652–0.951 | **0.013** |
| *Inner superior* | 0.838 | 0.695–1.010 | 0.064 |
| *Inner nasal* | 0.808 | 0.674–0.970 | **0.022** |
| *Inner inferior* | 0.822 | 0.681–0.993 | **0.042** |
| *Inner temporal* | 0.778 | 0.645–0.938 | **0.009** |
| Central field | 0.800 | 0.666–0.962 | **0.018** |

* Adjusted for age, sex, ethnicity, household income, Townsend deprivation index, education, smoking, drinking, body-mass index, use of antihypertensive, and lipid-lowering medication.

† Per-SD change of RPET, measured by SD-OCT using the ETDRS nine-pane grid.

‡ Bold indicates significant. Two-sided statistical tests were conducted, and no adjustments were made for multiple comparisons.

RPET = retinal pigment epithelium thickness; ETDRS = Early Treatment Diabetic Retinopathy Study; T2DM = type 2 diabetes mellitus; PRS = polygenetic risk score; AMD = age-related macular degeneration; HR = hazard ratio; CI = confidence interval; SD = standard deviation; SD-OCT = spectral-domain optical coherence tomography.

**Supplementary Table S4**

Sensitivity analyses on the association of average RPET with T2DM risk in UKB cohort estimated using multivariable CPH models.

| Risk of T2DM * | HR | 95% CI | P value † |
|---|---|---|---|
| **Overall population-I** | | | |
| Model 1 ‡ | 0.856 | 0.767–0.954 | **0.005** |
| Model 2 § | 0.868 | 0.779–0.968 | **0.011** |
| Model 3 ¶ | 0.849 | 0.757–0.951 | **0.005** |
| Model 4 # | 0.867 | 0.775–0.970 | **0.013** |
| Model 5 ** | 0.845 | 0.735–0.973 | **0.019** |
| **Stratified by PRS risk of AMD** | | | |
| Quartile 2 to Quartile 4 (Participants with low-moderate AMD risk) | | | |
| Model 1 ‡ | 0.835 | 0.734–0.950 | **0.006** |
| Model 2 § | 0.847 | 0.744–0.963 | **0.011** |
| Model 3 ¶ | 0.833 | 0.726–0.956 | **0.009** |
| Model 4 # | 0.833 | 0.727–0.954 | **0.009** |
| Model 5 ** | 0.792 | 0.666–0.941 | **0.008** |
| Quartile 1 (Participants with highest AMD risk) | | | |
| Model 1 ‡ | 0.900 | 0.731–1.107 | 0.319 |
| Model 2 § | 0.921 | 0.747–1.137 | 0.446 |
| Model 3 ¶ | 0.907 | 0.723–1.138 | 0.400 |
| Model 4 # | 0.940 | 0.762–1.161 | 0.568 |
| Model 5 ** | 0.969 | 0.757–1.241 | 0.802 |

* Per-SD change of RPET.

† Bold indicates significant. Two-sided statistical tests were conducted and no adjustments were made for multiple comparisons.

‡ Adjusted for age, sex, ethnicity, Townsend deprivation index, household income, education, smoking, drinking, body mass index, and use of antihypertensive and lipid-lowering medication.

§ Further adjusted for more comprehensive ethnicity classifications with further subdivisions for South Asian, East Asian, Black, and Mixed races.

¶ Further adjusted for biological ages.[1]

# Further adjusted for frailty score.[2]

** Further excluded cancer, cardiovascular diseases, and renal diseases.

RPET = retinal pigment epithelium thickness; T2DM = type 2 diabetes mellitus; HR = hazard ratio; CI = confidence interval; SD = standard deviation.

**Supplementary Table S5**

Metabolic biomarkers that were independently associated with average RPET after multiple testing correction.

| **Metabolic biomarkers** * | **β** † | **95%CI** | | $P_{raw}$ | $P_{FDR}$ ‡ |
|---|---|---|---|---|---|
| Triglycerides in Medium VLDL | -0.162 | -0.242 | -0.081 | $8.22×10^{-5}$ | 0.008 |
| Total Lipids in Medium VLDL | -0.160 | -0.240 | -0.080 | $9.31×10^{-5}$ | 0.008 |
| Triglycerides in Large VLDL | -0.161 | -0.243 | -0.078 | $1.41×10^{-4}$ | 0.008 |
| Total Lipids in Large VLDL | -0.157 | -0.240 | -0.075 | $1.79×10^{-4}$ | 0.008 |
| Concentration of Small VLDL Particles | -0.151 | -0.230 | -0.071 | $2.12×10^{-4}$ | 0.008 |
| Total Lipids in Small VLDL | -0.147 | -0.226 | -0.068 | $2.80×10^{-4}$ | 0.008 |
| Concentration of Medium VLDL Particles | -0.149 | -0.230 | -0.068 | $3.01×10^{-4}$ | 0.008 |
| Average Diameter for VLDL Particles | -0.204 | -0.316 | -0.092 | $3.65×10^{-4}$ | 0.008 |
| Cholesterol in Large VLDL | -0.147 | -0.228 | -0.065 | $4.22×10^{-4}$ | 0.008 |
| Phospholipids in Small LDL | -0.144 | -0.225 | -0.064 | $4.33×10^{-4}$ | 0.008 |
| Total Lipids in VLDL | -0.145 | -0.227 | -0.064 | $4.48×10^{-4}$ | 0.008 |
| Cholesteryl esters to total lipids ratio in large LDL | -0.159 | -0.248 | -0.070 | $4.58×10^{-4}$ | 0.008 |
| Cholesteryl Esters in Large VLDL | -0.143 | -0.224 | -0.063 | $4.90×10^{-4}$ | 0.008 |
| Triglycerides in Small VLDL | -0.142 | -0.222 | -0.062 | $4.95×10^{-4}$ | 0.008 |
| Phospholipids in Small VLDL | -0.142 | -0.221 | -0.062 | $5.21×10^{-4}$ | 0.008 |
| Concentration of Large VLDL Particles | -0.146 | -0.229 | -0.063 | $5.73×10^{-4}$ | 0.008 |
| Free Cholesterol in Large VLDL | -0.146 | -0.229 | -0.063 | $5.76×10^{-4}$ | 0.008 |
| Phospholipids in Medium VLDL | -0.141 | -0.222 | -0.060 | $6.18×10^{-4}$ | 0.009 |
| Triglycerides in VLDL | -0.141 | -0.223 | -0.058 | $8.30×10^{-4}$ | 0.010 |
| Total Lipids in Small LDL | -0.137 | -0.218 | -0.057 | $8.62×10^{-4}$ | 0.010 |
| Free Cholesterol in Medium VLDL | -0.138 | -0.220 | -0.057 | $8.73×10^{-4}$ | 0.010 |
| Phospholipids in Large VLDL | -0.141 | -0.224 | -0.058 | $8.81×10^{-4}$ | 0.010 |
| Ratio of triglycerides to phosphoglycerides | -0.146 | -0.232 | -0.059 | $9.39×10^{-4}$ | 0.010 |
| Phospholipids in VLDL | -0.134 | -0.216 | -0.053 | $1.17×10^{-3}$ | 0.012 |
| Cholesteryl Esters in Very Large VLDL | -0.135 | -0.217 | -0.054 | $1.19×10^{-3}$ | 0.012 |
| Free Cholesterol in VLDL | -0.134 | -0.216 | -0.053 | $1.22×10^{-3}$ | 0.012 |
| Cholesteryl Esters in Medium LDL | -0.130 | -0.210 | -0.050 | $1.42×10^{-3}$ | 0.013 |
| Concentration of VLDL Particles | -0.131 | -0.212 | -0.050 | $1.46×10^{-3}$ | 0.013 |
| Concentration of Small LDL Particles | -0.132 | -0.214 | -0.051 | $1.47×10^{-3}$ | 0.013 |
| Free Cholesterol in Small VLDL | -0.131 | -0.212 | -0.050 | $1.54×10^{-3}$ | 0.013 |
| Cholesterol in Very Large VLDL | -0.133 | -0.216 | -0.051 | $1.59×10^{-3}$ | 0.013 |
| Concentration of Medium LDL Particles | -0.131 | -0.212 | -0.049 | $1.66×10^{-3}$ | 0.013 |
| Tyrosine | 0.132 | 0.049 | 0.215 | $1.80×10^{-3}$ | 0.014 |
| Total Lipids in Very Large VLDL | -0.134 | -0.218 | -0.049 | $1.86×10^{-3}$ | 0.014 |
| Total Lipids in Medium LDL | -0.126 | -0.206 | -0.046 | $1.94×10^{-3}$ | 0.014 |
| Total Triglycerides | -0.128 | -0.210 | -0.046 | $2.14×10^{-3}$ | 0.014 |

| | | | | | |
|---|---|---|---|---|---|
| Cholesterol in Small LDL | -0.127 | -0.207 | -0.046 | $2.15\times10^{-3}$ | 0.014 |
| Cholesterol in Medium LDL | -0.125 | -0.205 | -0.045 | $2.18\times10^{-3}$ | 0.014 |
| Cholesteryl Esters in Small LDL | -0.126 | -0.206 | -0.045 | $2.30\times10^{-3}$ | 0.015 |
| Cholesterol in Small VLDL | -0.124 | -0.205 | -0.044 | $2.47\times10^{-3}$ | 0.015 |
| Concentration of Very Large VLDL Particles | -0.130 | -0.214 | -0.045 | $2.65\times10^{-3}$ | 0.016 |
| VLDL Cholesterol | -0.124 | -0.206 | -0.043 | $2.68\times10^{-3}$ | 0.016 |
| Ratio of apolipoprotein B to apolipoprotein A1 | -0.127 | -0.211 | -0.044 | $2.75\times10^{-3}$ | 0.016 |
| Triglycerides in Very Large VLDL | -0.130 | -0.215 | -0.045 | $2.76\times10^{-3}$ | 0.016 |
| Phospholipids in Medium LDL | -0.120 | -0.200 | -0.041 | $2.93\times10^{-3}$ | 0.016 |
| Concentration of LDL Particles | -0.123 | -0.204 | -0.041 | $3.11\times10^{-3}$ | 0.017 |
| Free Cholesterol in Very Large VLDL | -0.125 | -0.209 | -0.042 | $3.27\times10^{-3}$ | 0.017 |
| Phospholipids in Very Large VLDL | -0.125 | -0.209 | -0.041 | $3.69\times10^{-3}$ | 0.019 |
| Linoleic Acid | -0.118 | -0.197 | -0.038 | $3.86\times10^{-3}$ | 0.020 |
| Cholesteryl Esters in Small VLDL | -0.118 | -0.198 | -0.038 | $3.98\times10^{-3}$ | 0.020 |
| Phospholipids to total lipids ratio in small HDL | 0.116 | 0.036 | 0.195 | $4.34\times10^{-3}$ | 0.021 |
| Apolipoprotein B | -0.117 | -0.200 | -0.035 | $5.06\times10^{-3}$ | 0.024 |
| Isoleucine | 0.117 | 0.034 | 0.200 | $5.51\times10^{-3}$ | 0.026 |
| Cholesterol in Medium VLDL | -0.117 | -0.200 | -0.034 | $5.88\times10^{-3}$ | 0.027 |
| Free Cholesterol in Small LDL | -0.113 | -0.194 | -0.033 | $5.88\times10^{-3}$ | 0.027 |
| Cholesteryl Esters in LDL | -0.112 | -0.193 | -0.032 | $6.21\times10^{-3}$ | 0.028 |
| Concentration of Large LDL Particles | -0.112 | -0.193 | -0.031 | $6.94\times10^{-3}$ | 0.030 |
| Cholesteryl Esters in VLDL | -0.111 | -0.192 | -0.029 | $7.92\times10^{-3}$ | 0.034 |
| Triglycerides in Small LDL | -0.111 | -0.194 | -0.029 | $8.19\times10^{-3}$ | 0.035 |
| Total Lipids in LDL | -0.108 | -0.189 | -0.027 | $8.85\times10^{-3}$ | 0.037 |
| LDL Cholesterol | -0.106 | -0.187 | -0.024 | $1.08\times10^{-2}$ | 0.044 |
| Phospholipids in LDL | -0.105 | -0.185 | -0.024 | $1.12\times10^{-2}$ | 0.044 |
| Cholesteryl esters to total lipids ratio in medium LDL | -0.104 | -0.185 | -0.024 | $1.13\times10^{-2}$ | 0.044 |
| Leucine | 0.112 | 0.025 | 0.198 | $1.13\times10^{-2}$ | 0.044 |

* Adjusted for age, sex, ethnicity, assessment center, household income, Townsend deprivation index, education, smoking, drinking, body-mass index, intraocular pressure, spherical equivalent, use of lipid-lowering medications, and antihypertensive medications.

† Estimated with multiple linear regression models.

‡ Two-sided statistical tests were conducted, and BH method was employed to reduce false discovery rate for multiple tests.

CI = confidence interval; VLDL = very low-density lipoprotein; LDL = low-density

lipoprotein; HDL = high-density lipoprotein.

**Supplementary Table S6**

Multivariable-adjusted associations between the RPET-associated metabolites and T2DM risk.

| Metabolic biomarkers * | HR † | 95%CI | | $P_{raw}$ | $P_{FDR}$ ‡ |
|---|---|---|---|---|---|
| Triglycerides in Medium VLDL | 1.226 | 1.196 | 1.257 | $<1.0\times10^{-8}$ | $<1.0\times10^{-8}$ |
| Total Lipids in Medium VLDL | 1.072 | 1.043 | 1.103 | $1.11\times10^{-6}$ | $1.37\times10^{-6}$ |
| Triglycerides in Large VLDL | 1.266 | 1.236 | 1.295 | $<1.0\times10^{-8}$ | $<1.0\times10^{-8}$ |
| Total Lipids in Large VLDL | 1.264 | 1.232 | 1.296 | $<1.0\times10^{-8}$ | $<1.0\times10^{-8}$ |
| Concentration of Small VLDL Particles | 1.159 | 1.129 | 1.190 | $<1.0\times10^{-8}$ | $<1.0\times10^{-8}$ |
| Total Lipids in Small VLDL | 1.142 | 1.111 | 1.174 | $<1.0\times10^{-8}$ | $<1.0\times10^{-8}$ |
| Concentration of Medium VLDL Particles | 1.006 | 0.977 | 1.036 | 0.688 | 0.699 |
| Average Diameter for VLDL Particles | 1.438 | 1.386 | 1.492 | $<1.0\times10^{-8}$ | $<1.0\times10^{-8}$ |
| Cholesterol in Large VLDL | 1.195 | 1.163 | 1.227 | $<1.0\times10^{-8}$ | $<1.0\times10^{-8}$ |
| Phospholipids in Small LDL | 0.931 | 0.904 | 0.959 | $2.02\times10^{-6}$ | $2.39\times10^{-6}$ |
| Total Lipids in VLDL | 1.221 | 1.188 | 1.254 | $<1.0\times10^{-8}$ | $<1.0\times10^{-8}$ |
| Cholesteryl esters to total lipids ratio in large LDL | 0.963 | 0.951 | 0.974 | $<1.0\times10^{-8}$ | $<1.0\times10^{-8}$ |
| Cholesteryl Esters in Large VLDL | 1.113 | 1.082 | 1.145 | $<1.0\times10^{-8}$ | $<1.0\times10^{-8}$ |
| Triglycerides in Small VLDL | 1.273 | 1.243 | 1.303 | $<1.0\times10^{-8}$ | $<1.0\times10^{-8}$ |
| Phospholipids in Small VLDL | 1.019 | 0.990 | 1.050 | 0.201 | 0.222 |
| Concentration of Large VLDL Particles | 1.254 | 1.225 | 1.284 | $<1.0\times10^{-8}$ | $<1.0\times10^{-8}$ |
| Free Cholesterol in Large VLDL | 1.253 | 1.221 | 1.284 | $<1.0\times10^{-8}$ | $<1.0\times10^{-8}$ |
| Phospholipids in Medium VLDL | 0.990 | 0.961 | 1.020 | 0.522 | 0.548 |
| Triglycerides in VLDL | 1.309 | 1.276 | 1.342 | $<1.0\times10^{-8}$ | $<1.0\times10^{-8}$ |
| Total Lipids in Small LDL | 0.929 | 0.902 | 0.957 | $1.32\times10^{-6}$ | $1.59\times10^{-6}$ |
| Free Cholesterol in Medium VLDL | 0.922 | 0.894 | 0.951 | $3.15\times10^{-7}$ | $4.03\times10^{-7}$ |
| Phospholipids in Large VLDL | 1.273 | 1.242 | 1.304 | $<1.0\times10^{-8}$ | $<1.0\times10^{-8}$ |
| Ratio of triglycerides to phosphoglycerides | 1.439 | 1.401 | 1.478 | $<1.0\times10^{-8}$ | $<1.0\times10^{-8}$ |
| Phospholipids in VLDL | 1.176 | 1.144 | 1.208 | $<1.0\times10^{-8}$ | $<1.0\times10^{-8}$ |
| Cholesteryl Esters in Very Large VLDL | 1.142 | 1.111 | 1.175 | $<1.0\times10^{-8}$ | $<1.0\times10^{-8}$ |
| Free Cholesterol in VLDL | 1.122 | 1.092 | 1.154 | $<1.0\times10^{-8}$ | $<1.0\times10^{-8}$ |
| Cholesteryl Esters in Medium LDL | 0.923 | 0.896 | 0.950 | $9.52\times10^{-8}$ | $1.24\times10^{-7}$ |
| Concentration of VLDL Particles | 1.116 | 1.086 | 1.147 | $<1.0\times10^{-8}$ | $<1.0\times10^{-8}$ |
| Concentration of Small LDL Particles | 0.966 | 0.937 | 0.996 | $2.63\times10^{-2}$ | $3.01\times10^{-2}$ |
| Free Cholesterol in Small VLDL | 0.909 | 0.881 | 0.937 | $<1.0\times10^{-8}$ | $<1.0\times10^{-8}$ |
| Cholesterol in Very Large VLDL | 1.210 | 1.179 | 1.242 | $<1.0\times10^{-8}$ | $<1.0\times10^{-8}$ |
| Concentration of Medium LDL Particles | 0.913 | 0.885 | 0.942 | $<1.0\times10^{-8}$ | $<1.0\times10^{-8}$ |
| Tyrosine | 1.173 | 1.146 | 1.201 | $<1.0\times10^{-8}$ | $<1.0\times10^{-8}$ |
| Total Lipids in Very Large VLDL | 1.296 | 1.266 | 1.328 | $<1.0\times10^{-8}$ | $<1.0\times10^{-8}$ |
| Total Lipids in Medium LDL | 0.910 | 0.883 | 0.937 | $<1.0\times10^{-8}$ | $<1.0\times10^{-8}$ |
| Total Triglycerides | 1.307 | 1.275 | 1.340 | $<1.0\times10^{-8}$ | $<1.0\times10^{-8}$ |

| | | | | | |
|---|---|---|---|---|---|
| Cholesterol in Small LDL | 0.879 | 0.853 | 0.906 | $<1.0\times10^{-8}$ | $<1.0\times10^{-8}$ |
| Cholesterol in Medium LDL | 0.880 | 0.854 | 0.906 | $<1.0\times10^{-8}$ | $<1.0\times10^{-8}$ |
| Cholesteryl Esters in Small LDL | 0.926 | 0.899 | 0.955 | $6.96\times10^{-7}$ | $8.73\times10^{-7}$ |
| Cholesterol in Small VLDL | 0.968 | 0.939 | 0.998 | $3.68\times10^{-2}$ | $4.13\times10^{-2}$ |
| Concentration of Very Large VLDL Particles | 1.276 | 1.248 | 1.305 | $<1.0\times10^{-8}$ | $<1.0\times10^{-8}$ |
| VLDL Cholesterol | 1.018 | 0.988 | 1.048 | 0.243 | 0.264 |
| Ratio of apolipoprotein B to apolipoprotein A1 | 1.011 | 0.982 | 1.041 | 0.446 | 0.476 |
| Triglycerides in Very Large VLDL | 1.305 | 1.276 | 1.334 | $<1.0\times10^{-8}$ | $<1.0\times10^{-8}$ |
| Phospholipids in Medium LDL | 0.906 | 0.879 | 0.934 | $<1.0\times10^{-8}$ | $<1.0\times10^{-8}$ |
| Concentration of LDL Particles | 0.870 | 0.843 | 0.898 | $<1.0\times10^{-8}$ | $<1.0\times10^{-8}$ |
| Free Cholesterol in Very Large VLDL | 1.252 | 1.222 | 1.282 | $<1.0\times10^{-8}$ | $<1.0\times10^{-8}$ |
| Phospholipids in Very Large VLDL | 1.270 | 1.241 | 1.300 | $<1.0\times10^{-8}$ | $<1.0\times10^{-8}$ |
| Linoleic Acid | 0.899 | 0.874 | 0.924 | $<1.0\times10^{-8}$ | $<1.0\times10^{-8}$ |
| Cholesteryl Esters in Small VLDL | 1.005 | 0.975 | 1.035 | 0.765 | 0.765 |
| Phospholipids to total lipids ratio in small HDL | 1.096 | 1.067 | 1.125 | $<1.0\times10^{-8}$ | $<1.0\times10^{-8}$ |
| Apolipoprotein B | 0.869 | 0.843 | 0.896 | $<1.0\times10^{-8}$ | $<1.0\times10^{-8}$ |
| Isoleucine | 1.215 | 1.188 | 1.242 | $<1.0\times10^{-8}$ | $<1.0\times10^{-8}$ |
| Cholesterol in Medium VLDL | 0.775 | 0.750 | 0.800 | $<1.0\times10^{-8}$ | $<1.0\times10^{-8}$ |
| Free Cholesterol in Small LDL | 0.797 | 0.774 | 0.820 | $<1.0\times10^{-8}$ | $<1.0\times10^{-8}$ |
| Cholesteryl Esters in LDL | 0.840 | 0.817 | 0.865 | $<1.0\times10^{-8}$ | $<1.0\times10^{-8}$ |
| Concentration of Large LDL Particles | 0.838 | 0.812 | 0.865 | $<1.0\times10^{-8}$ | $<1.0\times10^{-8}$ |
| Cholesteryl Esters in VLDL | 0.938 | 0.909 | 0.967 | $4.15\times10^{-5}$ | $4.83\times10^{-5}$ |
| Triglycerides in Small LDL | 1.253 | 1.228 | 1.279 | $<1.0\times10^{-8}$ | $<1.0\times10^{-8}$ |
| Total Lipids in LDL | 0.838 | 0.814 | 0.862 | $<1.0\times10^{-8}$ | $<1.0\times10^{-8}$ |
| LDL Cholesterol | 0.811 | 0.789 | 0.834 | $<1.0\times10^{-8}$ | $<1.0\times10^{-8}$ |
| Phospholipids in LDL | 0.823 | 0.798 | 0.848 | $<1.0\times10^{-8}$ | $<1.0\times10^{-8}$ |
| Cholesteryl esters to total lipids ratio in medium LDL | 1.006 | 0.980 | 1.032 | 0.652 | 0.673 |
| Leucine | 1.236 | 1.208 | 1.265 | $<1.0\times10^{-8}$ | $<1.0\times10^{-8}$ |

* Adjusted for age, sex, ethnicity, household income, Townsend deprivation index, education, smoking, drinking, BMI, use of lipid-lowering medications, and antihypertensive medications.

† Estimated with CPH models.

‡ Two-sided statistical tests were conducted, and BH method was employed to reduce false discovery rate for multiple tests.

HR = hazard ratio; CI = confident interval; VLDL = very low-density lipoprotein; LDL = low-density lipoprotein; HDL = high-density lipoprotein.

**Supplementary Table S7**

Metabolic biomarkers that showed an inverse association between RPET alternations and T2DM risk.

| Metabolic biomarkers | Group |
|---|---|
| Triglycerides in Medium VLDL | Triglycerides |
| Total Lipids in Medium VLDL | Total lipids |
| Triglycerides in Large VLDL | Triglycerides |
| Total Lipids in Large VLDL | Total lipids |
| Concentration of Small VLDL Particles | Lipoprotein particle concentrations |
| Total Lipids in Small VLDL | Total lipids |
| Concentration of Medium VLDL Particles | Lipoprotein particle concentrations |
| Average Diameter for VLDL Particles | Lipoprotein particle sizes |
| Cholesterol in Large VLDL | Cholesterol |
| Total Lipids in VLDL | Total lipids |
| Cholesteryl Esters in Large VLDL | Cholesteryl esters |
| Triglycerides in Small VLDL | Triglycerides |
| Phospholipids in Small VLDL | Phospholipids |
| Concentration of Large VLDL Particles | Lipoprotein particle concentrations |
| Free Cholesterol in Large VLDL | Free cholesterol |
| Triglycerides in VLDL | Triglycerides |
| Phospholipids in Large VLDL | Phospholipids |
| Ratio of triglycerides to phosphoglycerides | Ratios |
| Phospholipids in VLDL | Phospholipids |
| Cholesteryl Esters in Very Large VLDL | Cholesteryl esters |
| Free Cholesterol in VLDL | Free cholesterol |
| Concentration of VLDL Particles | Lipoprotein particle concentrations |
| Cholesterol in Very Large VLDL | Cholesterol |
| Total Lipids in Very Large VLDL | Total lipids |
| Total Triglycerides | Triglycerides |
| Concentration of Very Large VLDL Particles | Lipoprotein particle concentrations |
| VLDL Cholesterol | Cholesterol |
| Ratio of apolipoprotein B to apolipoprotein A1 | Ratios |
| Triglycerides in Very Large VLDL | Triglycerides |
| Free Cholesterol in Very Large VLDL | Free cholesterol |
| Phospholipids in Very Large VLDL | Phospholipids |
| Cholesteryl Esters in Small VLDL | Cholesteryl esters |
| Triglycerides in Small LDL | Triglycerides |
| Cholesteryl esters to total lipids ratio in medium LDL | Ratios |

**Supplementary Table S8**

Multi-adjusted associations between the RPET-associated metabolites and ageing.

| Metabolic biomarkers * | HR † | 95%CI | | $P_{raw}$ | $P_{FDR}$ ‡ |
|---|---|---|---|---|---|
| VLDL Cholesterol | 0.073 | -0.006 | 0.152 | $7.11×10^{-2}$ | $8.27×10^{-2}$ |
| LDL Cholesterol | -0.488 | -0.570 | -0.407 | $<1.0×10^{-8}$ | $<1.0×10^{-8}$ |
| Total Triglycerides | 0.123 | 0.052 | 0.194 | $6.34×10^{-4}$ | $8.82×10^{-4}$ |
| Triglycerides in VLDL | -0.039 | -0.110 | 0.031 | 0.276 | 0.294 |
| Phospholipids in VLDL | 0.055 | -0.020 | 0.130 | 0.148 | 0.164 |
| Phospholipids in LDL | -0.465 | -0.548 | -0.382 | $<1.0×10^{-8}$ | $<1.0×10^{-8}$ |
| Cholesteryl Esters in VLDL | 0.099 | 0.018 | 0.180 | $1.64×10^{-2}$ | $2.01×10^{-2}$ |
| Cholesteryl Esters in LDL | -0.469 | -0.550 | -0.387 | $<1.0×10^{-8}$ | $<1.0×10^{-8}$ |
| Free Cholesterol in VLDL | 0.011 | -0.066 | 0.087 | 0.780 | 0.781 |
| Total Lipids in VLDL | 0.041 | -0.032 | 0.114 | 0.273 | 0.294 |
| Total Lipids in LDL | -0.411 | -0.493 | -0.330 | $<1.0×10^{-8}$ | $<1.0×10^{-8}$ |
| Concentration of VLDL Particles | 0.210 | 0.134 | 0.286 | $7.27×10^{-8}$ | $1.79×10^{-7}$ |
| Concentration of LDL Particles | -0.215 | -0.298 | -0.132 | $4.30×10^{-7}$ | $8.80×10^{-7}$ |
| Average Diameter for VLDL Particles | -0.354 | -0.425 | -0.284 | $<1.0×10^{-8}$ | $<1.0×10^{-8}$ |
| Apolipoprotein B | -0.088 | -0.172 | -0.004 | $3.98×10^{-2}$ | $4.71×10^{-2}$ |
| Linoleic Acid | -0.064 | -0.141 | 0.012 | $9.80×10^{-2}$ | $1.12×10^{-1}$ |
| Isoleucine | 0.524 | 0.459 | 0.589 | $<1.0×10^{-8}$ | $<1.0×10^{-8}$ |
| Leucine | 0.088 | 0.022 | 0.155 | $9.07×10^{-3}$ | $1.16×10^{-2}$ |
| Tyrosine | 1.024 | 0.960 | 1.087 | $<1.0×10^{-8}$ | $<1.0×10^{-8}$ |
| Concentration of Very Large VLDL Particles | -0.164 | -0.235 | -0.093 | $6.34×10^{-6}$ | $1.13×10^{-5}$ |
| Total Lipids in Very Large VLDL | -0.139 | -0.211 | -0.068 | $1.27×10^{-4}$ | $1.85×10^{-4}$ |
| Phospholipids in Very Large VLDL | -0.141 | -0.212 | -0.069 | $1.12×10^{-4}$ | $1.69×10^{-4}$ |
| Cholesterol in Very Large VLDL | -0.195 | -0.269 | -0.122 | $2.06×10^{-7}$ | $4.55×10^{-7}$ |
| Cholesteryl Esters in Very Large VLDL | -0.261 | -0.337 | -0.186 | $<1.0×10^{-8}$ | $<1.0×10^{-8}$ |
| Free Cholesterol in Very Large VLDL | -0.135 | -0.207 | -0.063 | $2.31×10^{-4}$ | $3.28×10^{-4}$ |
| Triglycerides in Very Large VLDL | -0.141 | -0.212 | -0.071 | $7.68×10^{-5}$ | $1.26×10^{-4}$ |
| Concentration of Large VLDL Particles | -0.184 | -0.256 | -0.113 | $4.40×10^{-7}$ | $8.80×10^{-7}$ |
| Total Lipids in Large VLDL | -0.198 | -0.270 | -0.126 | $6.53×10^{-8}$ | $1.67×10^{-7}$ |
| Phospholipids in Large VLDL | -0.189 | -0.261 | -0.117 | $2.78×10^{-7}$ | $5.93×10^{-7}$ |
| Cholesterol in Large VLDL | -0.169 | -0.243 | -0.096 | $6.81×10^{-6}$ | $1.18×10^{-5}$ |
| Cholesteryl Esters in Large VLDL | -0.117 | -0.192 | -0.043 | $2.10×10^{-3}$ | $2.80×10^{-3}$ |
| Free Cholesterol in Large VLDL | -0.222 | -0.295 | -0.149 | $<1.0×10^{-8}$ | $<1.0×10^{-8}$ |
| Triglycerides in Large VLDL | -0.241 | -0.311 | -0.171 | $<1.0×10^{-8}$ | $<1.0×10^{-8}$ |
| Concentration of Medium VLDL Particles | -0.235 | -0.314 | -0.156 | $<1.0×10^{-8}$ | $1.47×10^{-8}$ |
| Total Lipids in Medium VLDL | -0.187 | -0.264 | -0.111 | $1.55×10^{-6}$ | $2.93×10^{-6}$ |
| Phospholipids in Medium VLDL | -0.163 | -0.243 | -0.084 | $5.88×10^{-5}$ | $9.90×10^{-5}$ |

| | | | | | |
|---|---|---|---|---|---|
| Cholesterol in Medium VLDL | -0.167 | -0.251 | -0.084 | $9.05\times10^{-5}$ | $1.41\times10^{-4}$ |
| Free Cholesterol in Medium VLDL | -0.115 | -0.196 | -0.033 | $5.67\times10^{-3}$ | $7.41\times10^{-3}$ |
| Triglycerides in Medium VLDL | -0.192 | -0.263 | -0.121 | $1.27\times10^{-7}$ | $3.01\times10^{-7}$ |
| Concentration of Small VLDL Particles | 0.121 | 0.047 | 0.194 | $1.30\times10^{-3}$ | $1.77\times10^{-3}$ |
| Total Lipids in Small VLDL | 0.146 | 0.072 | 0.221 | $1.13\times10^{-4}$ | $1.69\times10^{-4}$ |
| Phospholipids in Small VLDL | 0.014 | -0.065 | 0.092 | 0.730 | 0.753 |
| Cholesterol in Small VLDL | 0.037 | -0.042 | 0.116 | 0.359 | 0.376 |
| Cholesteryl Esters in Small VLDL | 0.097 | 0.019 | 0.174 | $1.45\times10^{-2}$ | $1.83\times10^{-2}$ |
| Free Cholesterol in Small VLDL | -0.086 | -0.167 | -0.005 | $3.78\times10^{-2}$ | $4.56\times10^{-2}$ |
| Triglycerides in Small VLDL | 0.218 | 0.149 | 0.287 | $<1.0\times10^{-8}$ | $<1.0\times10^{-8}$ |
| Concentration of Large LDL Particles | -0.203 | -0.286 | -0.120 | $1.60\times10^{-6}$ | $2.93\times10^{-6}$ |
| Concentration of Medium LDL Particles | -0.270 | -0.351 | -0.190 | $<1.0\times10^{-8}$ | $<1.0\times10^{-8}$ |
| Total Lipids in Medium LDL | -0.562 | -0.642 | -0.482 | $<1.0\times10^{-8}$ | $<1.0\times10^{-8}$ |
| Phospholipids in Medium LDL | -0.659 | -0.739 | -0.578 | $<1.0\times10^{-8}$ | $<1.0\times10^{-8}$ |
| Cholesterol in Medium LDL | -0.606 | -0.687 | -0.526 | $<1.0\times10^{-8}$ | $<1.0\times10^{-8}$ |
| Cholesteryl Esters in Medium LDL | -0.558 | -0.638 | -0.478 | $<1.0\times10^{-8}$ | $<1.0\times10^{-8}$ |
| Concentration of Small LDL Particles | -0.067 | -0.149 | 0.015 | 0.107 | 0.121 |
| Total Lipids in Small LDL | -0.406 | -0.488 | -0.324 | $<1.0\times10^{-8}$ | $<1.0\times10^{-8}$ |
| Phospholipids in Small LDL | -0.161 | -0.242 | -0.081 | $8.44\times10^{-5}$ | $1.35\times10^{-4}$ |
| Cholesterol in Small LDL | -0.571 | -0.653 | -0.489 | $<1.0\times10^{-8}$ | $<1.0\times10^{-8}$ |
| Cholesteryl Esters in Small LDL | -0.536 | -0.618 | -0.454 | $<1.0\times10^{-8}$ | $<1.0\times10^{-8}$ |
| Free Cholesterol in Small LDL | -0.538 | -0.615 | -0.460 | $<1.0\times10^{-8}$ | $<1.0\times10^{-8}$ |
| Triglycerides in Small LDL | 0.201 | 0.132 | 0.271 | $1.20\times10^{-8}$ | $3.20\times10^{-8}$ |
| Ratio of triglycerides to phosphoglycerides | 0.010 | -0.062 | 0.083 | 0.781 | 0.781 |
| Ratio of apolipoprotein B to apolipoprotein A1 | -0.183 | -0.257 | -0.108 | $1.59\times10^{-6}$ | $2.93\times10^{-6}$ |
| Cholesteryl esters to total lipids ratio in large LDL | -0.629 | -0.696 | -0.562 | $<1.0\times10^{-8}$ | $<1.0\times10^{-8}$ |
| Cholesteryl esters to total lipids ratio in medium LDL | -0.180 | -0.247 | -0.112 | $2.00\times10^{-7}$ | $4.55\times10^{-7}$ |
| Phospholipids to total lipids ratio in small HDL | 0.974 | 0.907 | 1.040 | $<1.0\times10^{-8}$ | $<1.0\times10^{-8}$ |

* Adjusted for age, sex, ethnicity, household income, Townsend deprivation index, education, smoking, drinking, BMI, use of lipid-lowering medications, and antihypertensive medications.

† Estimated with CPH models.

‡ Two-sided statistical tests were conducted, and BH method was employed to reduce false discovery rate for multiple tests.

HR = hazard ratio; CI = confident interval; VLDL = very low-density lipoprotein; LDL = low-density lipoprotein; HDL = high-density lipoprotein.

**Supplementary Table S9**

Baseline characteristics of the Guangzhou Diabetic Eye Study (GDES) cohort population.

| Characteristic | GDES cohort | | | P value †‡ |
|---|---|---|---|---|
| | Overall population | Population-I | Population-II | |
| No. of subjects | 2373 | 1779 | 594 | - |
| Age, year | 64.47 (7.64) | 64.76 (7.55) | 63.61 (7.87) | **0.002** |
| Sex | | | | |
| *Female* | 1360 (57.3%) | 1013 (57.0%) | 347 (58.4%) | 0.570 |
| *Male* | 1012 (42.7%) | 765 (43.0%) | 247 (41.6%) | |
| Duration of diabetes, year | 8.86 (6.97) | 8.73 (6.93) | 9.24 (7.06) | 0.125 |
| Body-mass index, kg/m$^2$ | 24.57 (3.31) | 24.54 (3.39) | 24.64 (3.06) | 0.501 |
| Smoking status | | | | |
| *Ever/Current* | 1836 (84.5%) | 1415 (83.4%) | 421 (88.3%) | **0.012** |
| *Never* | 337 (15.5%) | 281 (16.6%) | 56 (11.7%) | |
| Alcohol drinking | | | | |
| *Ever/Current* | 1938 (89.5%) | 1498 (88.7%) | 440 (92.4%) | **0.023** |
| *Never* | 227 (10.5%) | 191 (11.3%) | 36 (7.6%) | |
| Hyperlipidemia | | | | |
| *No* | 1741 (73.4%) | 1413 (79.4%) | 328 (55.2%) | **<0.001** |
| *Yes* | 632 (26.6%) | 366 (20.6%) | 266 (44.8%) | |
| Use of insulin | | | | |
| *No* | 1930 (81.4%) | 1461 (82.2%) | 469 (79.0%) | 0.093 |
| *Yes* | 442 (18.6%) | 317 (17.8%) | 125 (21.0%) | |
| Systolic blood pressure, mmHg | 134.02 (18.57) | 135.07 (18.48) | 130.88 (18.47) | **<0.001** |
| HbA1c, % | 7.12 (1.42) | 7.19 (1.46) | 6.89 (1.26) | **<0.001** |

| | | | | |
|---|---|---|---|---|
| Low-density lipoprotein cholesterol, mmol/L | 3.04 (0.97) | 3.05 (0.99) | 3.01 (0.90) | 0.340 |
| High-density lipoprotein cholesterol, mmol/L | 1.29 (0.40) | 1.29 (0.41) | 1.27 (0.37) | 0.316 |
| Triglyceride, mmol/L | 2.44 (1.77) | 2.44 (1.78) | 2.41 (1.75) | 0.648 |
| Serum creatinine, mmol/L | 72.89 (24.84) | 72.59 (25.99) | 73.78 (20.97) | 0.314 |
| Urinary microalbumin, mg/L | 5.40 (26.33) | 5.50 (29.04) | 5.08 (15.38) | 0.736 |

† Student's t-test and chi-square test were used to compare continuous and categorical variables, respectively.

‡ Bold indicates significant. Two-sided statistical tests were conducted, and no adjustments were made for multiple comparisons.

**Supplementary Table S10**

Multivariable logistic models for evaluating association between RPET across all ETDRS subfields and diabetic retinopathy.

| RPET *† | Incident DR | | | | DR progression ‡ | | | |
|---|---|---|---|---|---|---|---|---|
| | OR | 95% CI | | P § | OR | 95% CI | | P § |
| **Total average** | 0.781 | 0.633 | 0.965 | **0.022** | 0.830 | 0.678 | 1.002 | 0.070 |
| **Outer average** | 0.786 | 0.628 | 0.983 | **0.035** | 0.846 | 0.685 | 1.045 | 0.121 |
| *Outer superior* | 0.767 | 0.600 | 0.981 | **0.035** | 0.763 | 0.600 | 0.969 | **0.026** |
| *Outer nasal* | 0.960 | 0.759 | 1.213 | 0.728 | 0.692 | 0.502 | 0.955 | **0.025** |
| *Outer inferior* | 0.890 | 0.663 | 1.195 | 0.437 | 0.783 | 0.537 | 1.141 | 0.203 |
| *Outer temporal* | 0.705 | 0.511 | 0.970 | **0.032** | 0.935 | 0.740 | 1.182 | 0.574 |
| **Inner average** | 0.736 | 0.588 | 0.922 | **0.008** | 0.738 | 0.592 | 0.920 | **0.007** |
| *Inner superior* | 0.832 | 0.658 | 1.051 | 0.124 | 0.714 | 0.560 | 0.909 | **0.006** |
| *Inner nasal* | 0.652 | 0.494 | 0.860 | **0.003** | 0.694 | 0.530 | 0.908 | **0.008** |
| *Inner inferior* | 0.887 | 0.682 | 1.151 | 0.370 | 0.783 | 0.573 | 1.070 | 0.125 |
| *Inner temporal* | 0.803 | 0.598 | 2.104 | 0.143 | 0.757 | 0.568 | 1.008 | 0.057 |
| *Central field* | 0.992 | 0.822 | 1.197 | 0.933 | 1.043 | 0.875 | 1.244 | 0.639 |

* Adjusted for age, sex, diabetes duration, HbA1c, body mass index, systolic blood pressure, smoking, drinking, and hyperlipidemia.

† Per-SD change of RPET, measured by SS-OCT using the ETDRS nine-pane grid.

‡ DR progression is defined as an increase in ETDRS grading score compared with baseline.

§ Bold indicates significant. Two-sided statistical tests were conducted, and no adjustments were made for multiple comparisons.

RPET = retinal pigment epithelium thickness; ETDRS = Early Treatment Diabetic Retinopathy Study; DR = diabetic retinopathy; CI = confidence interval, SS-OCT = swept-source optical coherence tomography.

**Supplementary Table S11**

Multivariable logistic models for evaluating association between RPET across all ETDRS subfields and fast progressor of renal function.

| RPET *† | Fast eGFR decline ‡§ | | | | Halving of eGFR § | | | |
|---|---|---|---|---|---|---|---|---|
| | OR | 95% CI | | P § | OR | 95% CI | | P ¶ |
| **Total average** | 0.839 | 0.703 | 0.999 | **0.049** | 0.271 | 0.111 | 0.665 | **0.004** |
| **Outer average** | 0.877 | 0.736 | 1.046 | 0.145 | 0.337 | 0.138 | 0.827 | **0.018** |
| *Outer superior* | 0.792 | 0.649 | 0.967 | **0.022** | 0.333 | 0.124 | 0.890 | **0.029** |
| *Outer nasal* | 0.739 | 0.557 | 0.978 | **0.035** | 0.648 | 0.189 | 2.217 | 0.489 |
| *Outer inferior* | 0.925 | 0.773 | 1.106 | 0.394 | 0.049 | 0.006 | 0.418 | **0.006** |
| *Outer temporal* | 0.936 | 0.797 | 1.100 | 0.422 | 0.152 | 0.039 | 0.586 | **0.006** |
| **Inner average** | 0.824 | 0.687 | 0.988 | **0.036** | 0.210 | 0.080 | 0.549 | **0.001** |
| *Inner superior* | 0.877 | 0.731 | 1.053 | 0.161 | 0.292 | 0.113 | 0.756 | **0.011** |
| *Inner nasal* | 0.767 | 0.619 | 0.952 | **0.016** | 0.281 | 0.096 | 0.824 | **0.021** |
| *Inner inferior* | 0.984 | 0.676 | 1.064 | 0.155 | 0.107 | 0.024 | 0.477 | **0.003** |
| *Inner temporal* | 0.909 | 0.753 | 1.099 | 0.325 | 0.095 | 0.026 | 0.353 | **<0.001** |
| *Central* | 0.992 | 0.829 | 1.186 | 0.927 | 0.127 | 0.039 | 0.414 | **0.001** |

* Adjusted for age, sex, diabetes duration, HbA1c, body mass index, systolic blood pressure, smoking, drinking, and hyperlipidemia.

† Per-SD change of RPET, measured by SS-OCT using the ETDRS nine-pane grid.

‡ GFR is estimated using the Chronic Kidney Disease-Epidemiology Collaboration (CKD-EPI) equation based on serum creatinine.[3]

§ Fast eGFR decline is defined as a decrease of 20% or more.[4,5] Halving of eGFR was defined as decline in eGFR by ≥ 50% from baseline.[6,7]

¶ Bold indicates significant. Two-sided statistical tests were conducted, and no adjustments were made for multiple comparisons.

RPET = retinal pigment epithelium thickness; ETDRS = Early Treatment Diabetic Retinopathy Study; eGFR = estimated glomerular filtration rate;

CI = confidence interval; SS-OCT = swept-source optical coherence tomography.

**Supplementary Table S12**

Multivariable logistic models for evaluating association between RPET across all ETDRS subfields and retinal capillary rarefaction.

| RPET *† | Fast retinal capillary rarefaction in macula ‡ | | | | Fast retinal capillary rarefaction in ONH ‡ | | | |
|---|---|---|---|---|---|---|---|---|
| | OR | 95% CI | | P § | OR | 95% CI | | P § |
| **Total average** | 0.783 | 0.611 | 1.004 | 0.054 | 0.825 | 0.586 | 1.162 | 0.272 |
| **Outer average** | 0.739 | 0.564 | 0.969 | **0.029** | 0.856 | 0.589 | 1.244 | 0.414 |
| *Outer superior* | 1.142 | 0.922 | 1.415 | 0.221 | 0.896 | 0.592 | 1.357 | 0.603 |
| *Outer nasal* | 0.987 | 0.758 | 1.285 | 0.924 | 0.736 | 0.417 | 1.302 | 0.293 |
| *Outer inferior* | 0.872 | 0.637 | 1.192 | 0.391 | 0.820 | 0.444 | 1.516 | 0.528 |
| *Outer temporal* | 0.743 | 0.557 | 0.992 | **0.044** | 0.733 | 0.427 | 1.259 | 0.260 |
| **Inner average** | 0.891 | 0.689 | 1.155 | 0.385 | 0.758 | 0.520 | 1.104 | 0.150 |
| *Inner superior* | 0.934 | 0.744 | 1.174 | 0.557 | 0.624 | 0.410 | 0.949 | **0.028** |
| *Inner nasal* | 0.946 | 0.742 | 1.208 | 0.657 | 0.866 | 0.572 | 1.310 | 0.494 |
| *Inner inferior* | 0.760 | 0.533 | 1.084 | 0.130 | 0.780 | 0.450 | 1.355 | 0.379 |
| *Inner temporal* | 0.855 | 0.676 | 1.081 | 0.191 | 0.560 | 0.331 | 0.950 | **0.032** |
| *Central* | 0.828 | 0.622 | 1.102 | 0.196 | 0.934 | 0.666 | 1.310 | 0.692 |

* Adjusted for age, sex, diabetes duration, HbA1c, body mass index, systolic blood pressure, smoking, drinking, and history of hyperlipidemia.

† Per-SD change of RPET, measured by SS-OCT using the ETDRS nine-pane grid.

‡ Fast capillary rarefaction is defined as the longitudinal rates of vessel density decline of retinal deep capillary plexus in macular or optic nerve head regions measured by SS-OCT-angiography being in the first quartile, respectively.

§ Bold indicates significant. Two-sided statistical tests were conducted, and no adjustments were made for multiple comparisons.

ONH = optic nerve head; RPET = retinal pigment epithelium thickness; ETDRS = Early Treatment Diabetic Retinopathy Study; CI = confidence

interval; SS-OCT = swept-source optical coherence tomography.

**Supplementary Table S13**

RPET-associated metabolites identified using LC/MS assay in the Guangzhou Diabetic Eye Study (GDES) cohort after adjusting for other factors.

| Metabolites * | Group | β † | 95%CI | | $P_{raw}$ | $P_{FDR}$ ‡ |
|---|---|---|---|---|---|---|
| γ-Glu-Met | Amino acid and Its metabolites | 2.855 | 2.147 | 3.563 | $<1.0\times10^{-8}$ | $<1.0\times10^{-8}$ |
| L-Arginine | Amino acid and Its metabolites | 2.607 | 1.882 | 3.332 | $<1.0\times10^{-8}$ | $<1.0\times10^{-8}$ |
| Tryptamine | Tryptamines,Cholines,Pigments | 2.327 | 1.590 | 3.065 | $<1.0\times10^{-8}$ | $2.72\times10^{-7}$ |
| Val-Ala | Amino acid and Its metabolites | 2.247 | 1.525 | 2.969 | $<1.0\times10^{-8}$ | $3.57\times10^{-7}$ |
| Met-Glu | Amino acid and Its metabolites | 2.270 | 1.535 | 3.006 | $<1.0\times10^{-8}$ | $4.07\times10^{-7}$ |
| Piperidine | Heterocyclic compounds | 2.314 | 1.532 | 3.096 | $1.08\times10^{-8}$ | $1.38\times10^{-6}$ |
| Azelaic Acid | Organic acid and Its derivatives | 2.091 | 1.366 | 2.816 | $2.44\times10^{-8}$ | $2.38\times10^{-6}$ |
| Glu-Met | Amino acid and Its metabolites | 2.112 | 1.375 | 2.850 | $3.00\times10^{-8}$ | $2.67\times10^{-6}$ |
| N-Propionylglycine | Amino acid and Its metabolites | 2.204 | 1.434 | 2.974 | $3.11\times10^{-8}$ | $2.72\times10^{-6}$ |
| 2-(Formylamino)Benzoic Acid | Benzene and substituted derivatives | 1.989 | 1.258 | 2.721 | $1.39\times10^{-7}$ | $1.07\times10^{-5}$ |
| 2'-O-methylcytidine | Nucleotide and Its metabolites | 1.965 | 1.231 | 2.698 | $2.11\times10^{-7}$ | $1.51\times10^{-5}$ |
| 3-Hydroxy-L-phenylalanine | Amino acid and Its metabolites | 1.854 | 1.120 | 2.588 | $9.72\times10^{-7}$ | $6.37\times10^{-5}$ |
| DL-O-tyrosine | Amino acid and Its metabolites | 1.777 | 1.032 | 2.521 | $3.63\times10^{-6}$ | $2.19\times10^{-4}$ |
| D-Alloisoleucine | Amino acid and Its metabolites | 1.747 | 0.959 | 2.534 | $1.62\times10^{-5}$ | $8.48\times10^{-4}$ |
| 5,6-Dimethylbenzimidazole | Heterocyclic compounds | 1.654 | 0.903 | 2.405 | $1.85\times10^{-5}$ | $9.45\times10^{-4}$ |
| L-Methionine | Amino acid and Its metabolites | 1.653 | 0.891 | 2.416 | $2.47\times10^{-5}$ | $1.19\times10^{-3}$ |
| Indoleacetaldehyde | Heterocyclic compounds | 1.574 | 0.829 | 2.319 | $3.95\times10^{-5}$ | $1.66\times10^{-3}$ |
| 9,10-Epoxy-18-hydroxyoctadecanoic acid | FA | 1.531 | 0.801 | 2.261 | $4.53\times10^{-5}$ | $1.81\times10^{-3}$ |
| Trp-Gly | Amino acid and Its metabolites | 1.603 | 0.837 | 2.368 | $4.64\times10^{-5}$ | $1.84\times10^{-3}$ |
| Glycolithocholic acid | Bile acids | 1.515 | 0.782 | 2.248 | $5.75\times10^{-5}$ | $2.09\times10^{-3}$ |
| 2'-O-methyluridine | Nucleotide and Its metabolites | 1.561 | 0.803 | 2.320 | $6.21\times10^{-5}$ | $2.18\times10^{-3}$ |
| Melatonin | Hormones and hormone related compounds | 1.534 | 0.788 | 2.279 | $6.23\times10^{-5}$ | $2.19\times10^{-3}$ |

| | | | | | | |
|---|---|---|---|---|---|---|
| N-Acetyl-L-Leucine | Amino acid and Its metabolites | 1.658 | 0.849 | 2.467 | $6.62\times10^{-5}$ | $2.26\times10^{-3}$ |
| gamma-Glu-Phe | Amino acid and Its metabolites | 1.593 | 0.796 | 2.390 | $1.00\times10^{-4}$ | $3.10\times10^{-3}$ |
| 12,13-DiHOME | FA | 1.453 | 0.722 | 2.184 | $1.09\times10^{-4}$ | $3.27\times10^{-3}$ |
| 9,10-DiHOME | FA | 1.449 | 0.718 | 2.180 | $1.15\times10^{-4}$ | $3.40\times10^{-3}$ |
| N-acetylornithine | Amino acid and Its metabolites | 1.481 | 0.730 | 2.231 | $1.22\times10^{-4}$ | $3.54\times10^{-3}$ |
| Gly-Phe | Amino acid and Its metabolites | 1.437 | 0.699 | 2.174 | $1.49\times10^{-4}$ | $4.14\times10^{-3}$ |
| Glu-Tyr | Amino acid and Its metabolites | 1.462 | 0.705 | 2.220 | $1.70\times10^{-4}$ | $4.58\times10^{-3}$ |
| Phe-Hyp | Amino acid and Its metabolites | 1.410 | 0.673 | 2.146 | $1.94\times10^{-4}$ | $5.04\times10^{-3}$ |
| Phe-Phe | Amino acid and Its metabolites | 1.417 | 0.670 | 2.165 | $2.23\times10^{-4}$ | $5.55\times10^{-3}$ |
| Pro-Asp | Amino acid and Its metabolites | 1.398 | 0.658 | 2.138 | $2.33\times10^{-4}$ | $5.71\times10^{-3}$ |
| Glu-Leu | Amino acid and Its metabolites | 1.455 | 0.676 | 2.234 | $2.73\times10^{-4}$ | $6.40\times10^{-3}$ |
| Phe-Gly | Amino acid and Its metabolites | 1.385 | 0.641 | 2.129 | $2.86\times10^{-4}$ | $6.60\times10^{-3}$ |
| L-Lysine | Amino acid and Its metabolites | 1.360 | 0.624 | 2.096 | $3.20\times10^{-4}$ | $7.16\times10^{-3}$ |
| L-Serine | Amino acid and Its metabolites | 1.383 | 0.629 | 2.137 | $3.53\times10^{-4}$ | $7.69\times10^{-3}$ |
| N-Amidino-L-Aspartate | Amino acid and Its metabolites | 1.356 | 0.609 | 2.103 | $4.04\times10^{-4}$ | $8.42\times10^{-3}$ |
| Multifidol | Benzene and substituted derivatives | 1.334 | 0.594 | 2.073 | $4.40\times10^{-4}$ | $8.90\times10^{-3}$ |
| Urocanic Acid | Organic acid and Its derivatives | 1.338 | 0.595 | 2.080 | $4.46\times10^{-4}$ | $8.98\times10^{-3}$ |
| PI (15:0/2:0) | GP | 1.321 | 0.577 | 2.064 | $5.35\times10^{-4}$ | $1.01\times10^{-2}$ |
| Caffeic Acid | Organic acid and Its derivatives | 1.303 | 0.570 | 2.037 | $5.37\times10^{-4}$ | $1.01\times10^{-2}$ |
| Hydroxyurea | Others | 1.310 | 0.572 | 2.049 | $5.41\times10^{-4}$ | $1.01\times10^{-2}$ |
| 5'-Deoxy-5'-(Methylthio) Adenosine | Nucleotide and Its metabolites | 1.318 | 0.556 | 2.080 | $7.45\times10^{-4}$ | $1.32\times10^{-2}$ |
| Quinmerac | Benzene and substituted derivatives | -1.460 | -2.306 | -0.614 | $7.66\times10^{-4}$ | $1.35\times10^{-2}$ |
| Proline betaine | Amino acid and Its metabolites | 1.278 | 0.528 | 2.028 | $8.89\times10^{-4}$ | $1.52\times10^{-2}$ |
| L-Ornithine | Amino acid and Its metabolites | 1.256 | 0.519 | 1.994 | $8.96\times10^{-4}$ | $1.53\times10^{-2}$ |
| L-Glutamine | Amino acid and Its metabolites | 1.243 | 0.507 | 1.980 | $9.98\times10^{-4}$ | $1.66\times10^{-2}$ |
| N, N-Bis(2-hydroxyethyl) dodecanamide | FA | 1.238 | 0.496 | 1.979 | $1.14\times10^{-3}$ | $1.84\times10^{-2}$ |

| | | | | | | |
|---|---|---|---|---|---|---|
| Trolox | Benzene and substituted derivatives | 1.235 | 0.491 | 1.978 | $1.20\times10^{-3}$ | $1.91\times10^{-2}$ |
| Asp-Leu | Amino acid and Its metabolites | 1.220 | 0.482 | 1.958 | $1.27\times10^{-3}$ | $2.00\times10^{-2}$ |
| 4-Acetylaminobenzoic acid | Organic acid and Its derivatives | 1.205 | 0.469 | 1.940 | $1.40\times10^{-3}$ | $2.15\times10^{-2}$ |
| N-(2-hydroxyethyl)-3-pyridinecarboxamide | Heterocyclic compounds | 1.307 | 0.502 | 2.112 | $1.53\times10^{-3}$ | $2.31\times10^{-2}$ |
| Creatine phosphate | Nucleotide and Its metabolites | 1.194 | 0.446 | 1.943 | $1.86\times10^{-3}$ | $2.71\times10^{-2}$ |
| Phe-Val | Amino acid and Its metabolites | 1.197 | 0.446 | 1.948 | $1.87\times10^{-3}$ | $2.72\times10^{-2}$ |
| 2-Aminophenol | Benzene and substituted derivatives | 1.157 | 0.421 | 1.892 | $2.14\times10^{-3}$ | $3.06\times10^{-2}$ |
| Hippuric Acid | Organic acid and Its derivatives | 1.120 | 0.385 | 1.855 | $2.95\times10^{-3}$ | $4.01\times10^{-2}$ |
| L-Asparagine Anhydrous | Amino acid and Its metabolites | 1.162 | 0.394 | 1.929 | $3.14\times10^{-3}$ | $4.21\times10^{-2}$ |
| Phenethylamine | Alcohol and amines | 1.175 | 0.384 | 1.966 | $3.75\times10^{-3}$ | $4.85\times10^{-2}$ |

* Adjusted for age, sex duration of diabetes, HbA1c, body-mass index, systolic blood pressure, smoking, drinking, and hyperlipidemia.

† Estimated with multiple linear regression models.

‡ Two-sided statistical tests were conducted, and BH method was employed to reduce false discovery rate for multiple tests.

**Supplementary Table S14**

STROBE Statement—Checklist of items that should be included in reports of cohort studies.

| | Item No | Recommendation | Page No |
|---|---|---|---|
| **Title and abstract** | 1 | (*a*) Indicate the study's design with a commonly used term in the title or the abstract | 1 |
| | | (*b*) Provide in the abstract an informative and balanced summary of what was done and what was found | 3 |
| **Introduction** | | | |
| Background/rationale | 2 | Explain the scientific background and rationale for the investigation being reported | 4 |
| Objectives | 3 | State specific objectives, including any prespecified hypotheses | 4 |
| **Methods** | | | |
| Study design | 4 | Present key elements of study design early in the paper | 5 |
| Setting | 5 | Describe the setting, locations, and relevant dates, including periods of recruitment, exposure, follow-up, and data collection | 14 |
| Participants | 6 | (*a*) Give the eligibility criteria, and the sources and methods of selection of participants. Describe methods of follow-up | 15 |
| | | (*b*) For matched studies, give matching criteria and number of exposed and unexposed | x |
| Variables | 7 | Clearly define all outcomes, exposures, predictors, potential confounders, and effect modifiers. Give diagnostic criteria, if applicable | 16 |
| Data sources/ measurement | 8* | For each variable of interest, give sources of data and details of methods of assessment (measurement). Describe comparability of assessment methods if there is more than one group | 17 |
| Bias | 9 | Describe any efforts to address potential sources of bias | 19 |
| Study size | 10 | Explain how the study size was arrived at | 15 |
| Quantitative variables | 11 | Explain how quantitative variables were handled in the analyses. If applicable, describe which groupings were chosen and why | 19 |
| Statistical methods | 12 | (*a*) Describe all statistical methods, including those used to control for confounding | 19 |
| | | (*b*) Describe any methods used to examine subgroups and interactions | 19 |
| | | (*c*) Explain how missing data were addressed | 19 |
| | | (*d*) If applicable, explain how loss to follow-up was addressed | 19 |
| | | (*e*) Describe any sensitivity analyses | 19 |

**Results**

| | | | |
|---|---|---|---|
| Participants | 13* | (a) Report numbers of individuals at each stage of study—eg numbers potentially eligible, examined for eligibility, confirmed eligible, included in the study, completing follow-up, and analysed | 5 |
| | | (b) Give reasons for non-participation at each stage | 15 |
| | | (c) Consider use of a flow diagram | 5 |
| Descriptive data | 14* | (a) Give characteristics of study participants (eg demographic, clinical, social) and information on exposures and potential confounders | 5 |
| | | (b) Indicate number of participants with missing data for each variable of interest | 5 |
| | | (c) Summarise follow-up time (eg, average and total amount) | 6 |
| Outcome data | 15* | Report numbers of outcome events or summary measures over time | 6 |

| | | | |
|---|---|---|---|
| Main results | 16 | (*a*) Give unadjusted estimates and, if applicable, confounder-adjusted estimates and their precision (eg, 95% confidence interval). Make clear which confounders were adjusted for and why they were included | 5 |
| | | (*b*) Report category boundaries when continuous variables were categorized | 5 |
| | | (*c*) If relevant, consider translating estimates of relative risk into absolute risk for a meaningful time period | × |
| Other analyses | 17 | Report other analyses done—eg analyses of subgroups and interactions, and sensitivity analyses | 5 |

**Discussion**

| | | | |
|---|---|---|---|
| Key results | 18 | Summarise key results with reference to study objectives | 8 |
| Limitations | 19 | Discuss limitations of the study, taking into account sources of potential bias or imprecision. Discuss both direction and magnitude of any potential bias | 13 |
| Interpretation | 20 | Give a cautious overall interpretation of results considering objectives, limitations, multiplicity of analyses, results from similar studies, and other relevant evidence | 14 |
| Generalisability | 21 | Discuss the generalisability (external validity) of the study results | 13 |

**Other information**

| | | | |
|---|---|---|---|
| Funding | 22 | Give the source of funding and the role of the funders for the present study and, if applicable, for the original study on which the present article is based | 29 |

**Supplementary Table S15**

STARD guidelines.

| Section & Topic | No | Item | Reported on page # |
|---|---|---|---|
| **TITLE OR ABSTRACT** | | | 3 |
| | 1 | Identification as a study of diagnostic accuracy using at least one measure of accuracy<br>(such as sensitivity, specificity, predictive values, or AUC) | 3 |
| **ABSTRACT** | | | 3 |
| | 2 | Structured summary of study design, methods, results, and conclusions<br>(for specific guidance, see STARD for Abstracts) | 3 |
| **INTRODUCTION** | | | 4 |
| | 3 | Scientific and clinical background, including the intended use and clinical role of the index test | 4 |
| | 4 | Study objectives and hypotheses | 4 |
| **METHODS** | | | 14 |
| *Study design* | 5 | Whether data collection was planned before the index test and reference standard<br>were performed (prospective study) or after (retrospective study) | 14 |
| *Participants* | 6 | Eligibility criteria | 15 |
| | 7 | On what basis potentially eligible participants were identified<br>(such as symptoms, results from previous tests, inclusion in registry) | 14 |
| | 8 | Where and when potentially eligible participants were identified<br>(setting, location and dates) | 14 |
| | 9 | Whether participants formed a consecutive, random or convenience series | 14 |
| *Test methods* | 10a | Index test, in sufficient detail to allow replication | 15 |
| | 10b | Reference standard, in sufficient detail to allow replication | 17 |
| | 11 | Rationale for choosing the reference standard (if alternatives exist) | 16 |
| | 12a | Definition of and rationale for test positivity cut-offs or result categories<br>of the index test, distinguishing pre-specified from exploratory | 20 |
| | 12b | Definition of and rationale for test positivity cut-offs or result categories | 20 |

| | | | |
|---|---|---|---|
| | | of the reference standard, distinguishing pre-specified from exploratory | |
| | 13a | Whether clinical information and reference standard results were available to the performers/readers of the index test | 16 |
| | 13b | Whether clinical information and index test results were available to the assessors of the reference standard | 16 |
| *Analysis* | 14 | Methods for estimating or comparing measures of diagnostic accuracy | 20 |
| | 15 | How indeterminate index test or reference standard results were handled | 16 |
| | 16 | How missing data on the index test and reference standard were handled | 20 |
| | 17 | Any analyses of variability in diagnostic accuracy, distinguishing pre-specified from exploratory | NA |
| | 18 | Intended sample size and how it was determined | 15 |
| **RESULTS** | | | 5 |
| *Participants* | 19 | Flow of participants, using a diagram | 5 |
| | 20 | Baseline demographic and clinical characteristics of participants | 5 |
| | 21a | Distribution of severity of disease in those with the target condition | 5 |
| | 21b | Distribution of alternative diagnoses in those without the target condition | 5 |
| | 22 | Time interval and any clinical interventions between index test and reference standard | 15 |
| *Test results* | 23 | Cross tabulation of the index test results (or their distribution) by the results of the reference standard | 7 |
| | 24 | Estimates of diagnostic accuracy and their precision (such as 95% confidence intervals) | 7 |
| | 25 | Any adverse events from performing the index test or the reference standard | 15 |
| **DISCUSSION** | | | 8 |
| | 26 | Study limitations, including sources of potential bias, statistical uncertainty, and generalisability | 13 |
| | 27 | Implications for practice, including the intended use and clinical role of the index test | 14 |
| **OTHER INFORMATION** | | | 22 |

| | | | |
|---|---|---|---|
| | 28 | Registration number and name of registry | 14 |
| | 29 | Where the full study protocol can be accessed | 14 |
| | 30 | Sources of funding and other support; role of funders | 29 |

## SUPPLEMENTARY FIGURES

### Supplementary Figure S1

Sensitivity analysis of constructing metabolic state model and combined model incorporating only metabolic biomarkers that were associated with both thinner RPET and increased T2DM risk for incident T2DM (n=5,714).

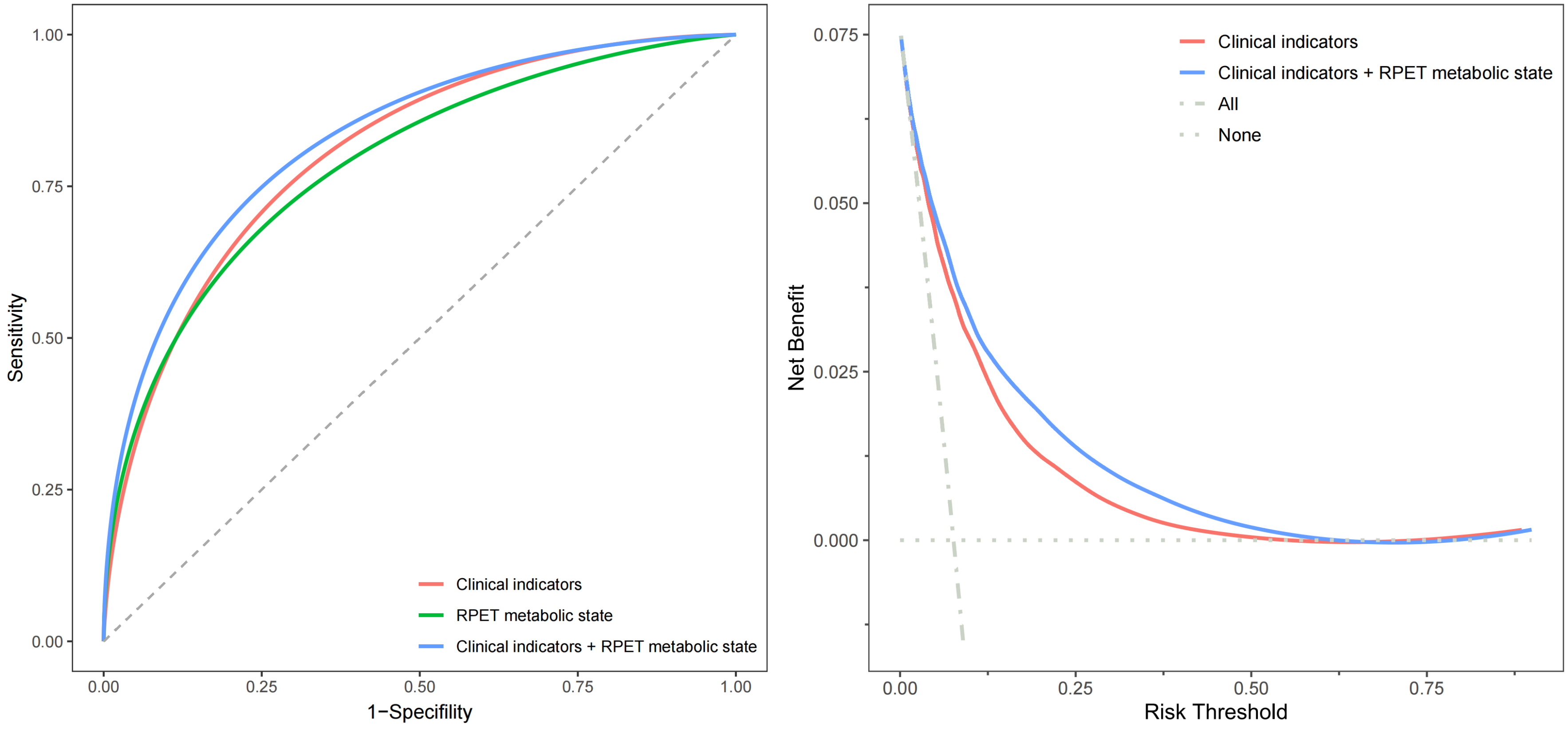

**Supplementary Figure S2**

Sensitivity analysis of constructing metabolic state model and combined model incorporating only metabolic biomarkers that were independent of ageing for incident T2DM (n=5,714).

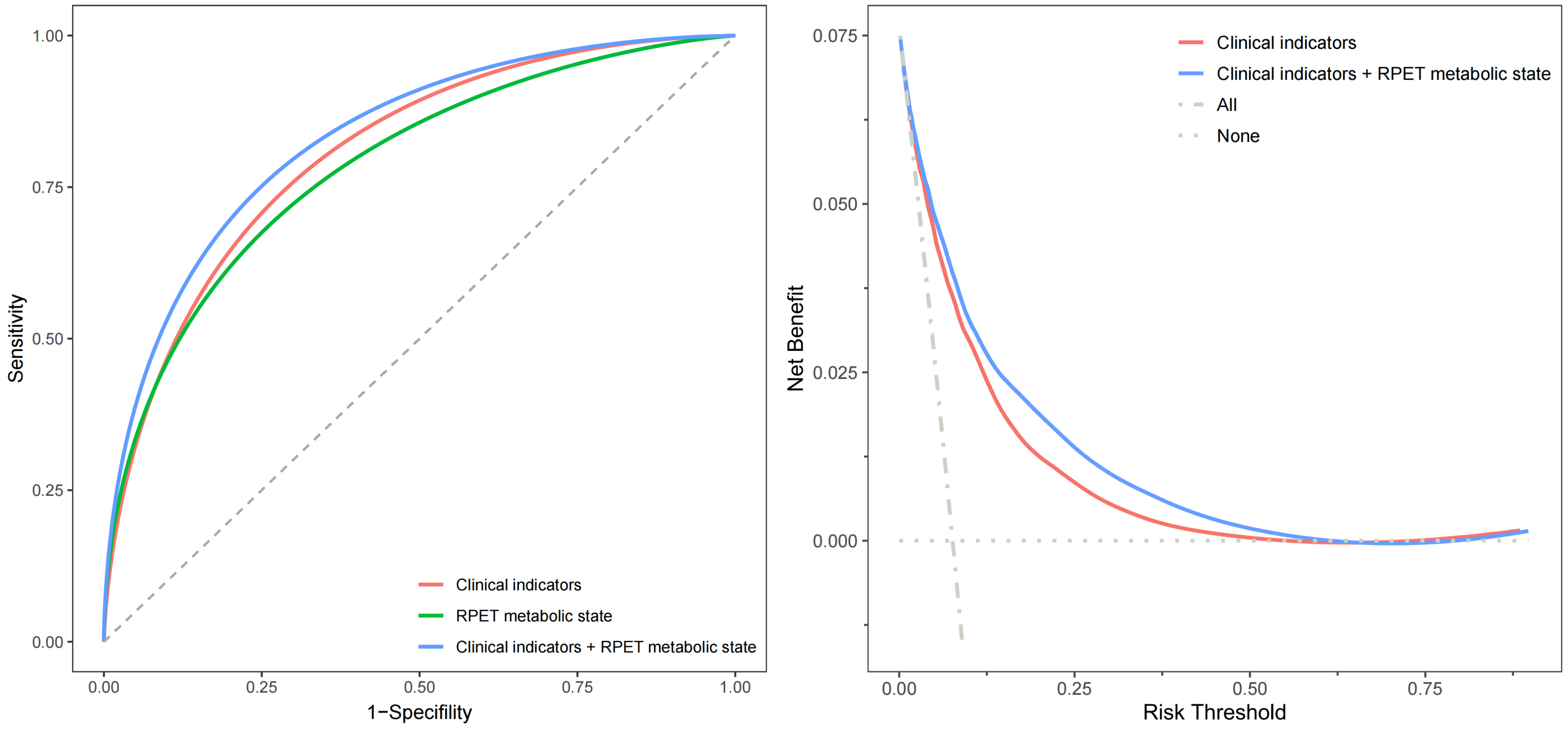

**Supplementary Figure S3**

Added predictability and clinical utility of RPET metabolic fingerprints for stratifying T2DM adverse microvascular phenotypes (incident DR, n=161; DR progression, n=160; rapid eGFR decrease, n=201; rapid macular blood flow decrease, n=46; rapid parafoveal blood flow decrease, n=45).

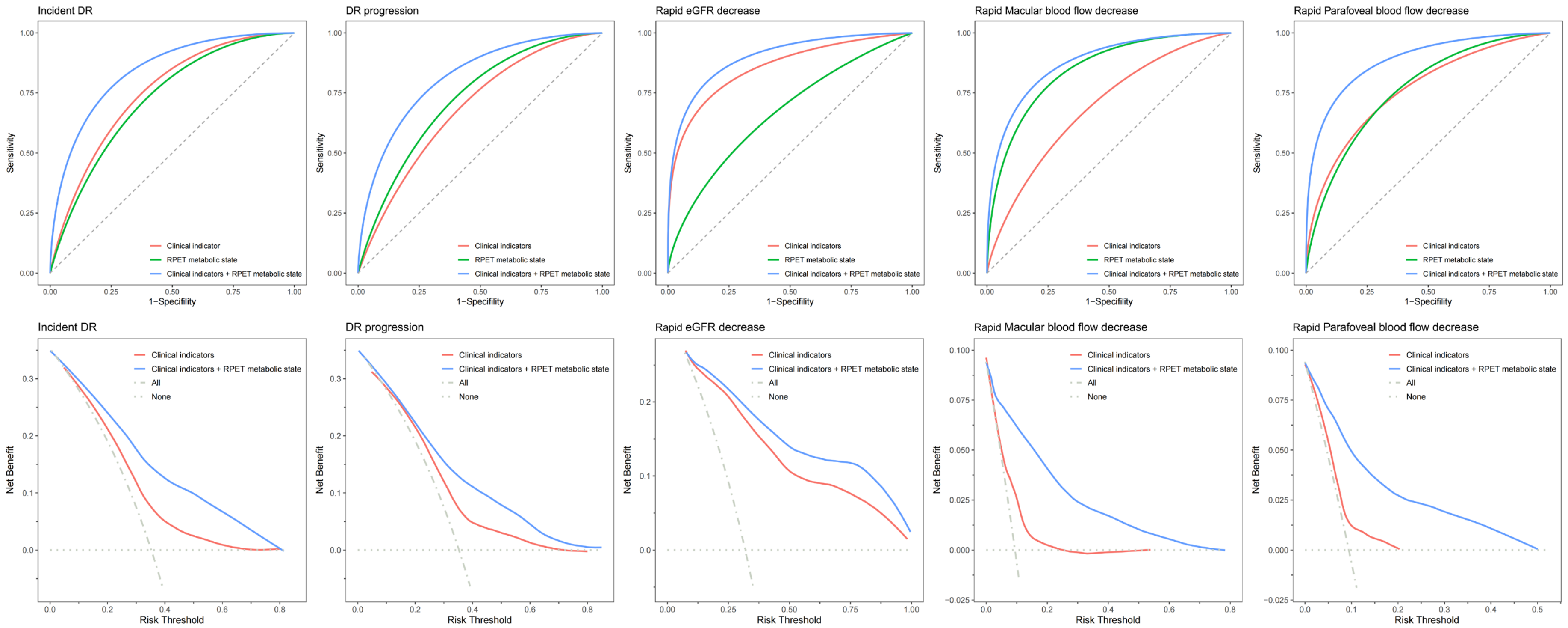

**SUPPLEMENTARY REFERENCES**


1. Yang, G. et al. Association of Unhealthy Lifestyle and Childhood Adversity With Acceleration of Aging Among UK Biobank Participants. *Jama Netw. Open*. **5**, e2230690 (2022).
2. Fried, L. P. et al. Frailty in older adults: evidence for a phenotype. *J. Gerontol. Ser. A-Biol. Sci. Med. Sci.* **56**, M146-M156 (2001).
3. Levey, A. S. & Stevens, L. A. Estimating GFR using the CKD Epidemiology Collaboration (CKD-EPI) creatinine equation: more accurate GFR estimates, lower CKD prevalence estimates, and better risk predictions. *Am. J. Kidney. Dis.* **55**, 622-627 (2010).
4. Yang, W. et al. Association of kidney disease outcomes with risk factors for CKD: findings from the Chronic Renal Insufficiency Cohort (CRIC) study. *Am. J. Kidney. Dis.* **63**, 236-243 (2014).
5. Heinzel, A. et al. Validation of Plasma Biomarker Candidates for the Prediction of eGFR Decline in Patients With Type 2 Diabetes. *Diabetes Care*. **41**, 1947-1954 (2018).
6. Wen, D. et al. Metabolite profiling of CKD progression in the chronic renal insufficiency cohort study. *Jci Insight*. **7**, (2022).
7. Anderson, A. H. et al. Novel Risk Factors for Progression of Diabetic and Nondiabetic CKD: Findings From the Chronic Renal Insufficiency Cohort (CRIC) Study. *Am. J. Kidney. Dis.* **77**, 56-73 (2021).
