# Supplementary material for: Metabolic fingerprinting on retinal pigment epithelium thickness for individualized risk stratification of type 2 diabetes mellitus
Source: Nat Commun. 2023 Oct 18;14:6573. doi: 10.1038/s41467-023-42404-1 (PMC10585002; doi:10.1038/s41467-023-42404-1)
Supplement: Supplementary file 3 — Reporting Summary [file 41467_2023_42404_MOESM3_ESM.pdf]

nature portfolio

Corresponding author(s): Wei Wang

Last updated by author(s): Oct 9, 2023

Reporting Summary

Nature Portfolio wishes to improve the reproducibility of the work that we publish. This form provides structure for consistency and transparency in reporting. For further information on Nature Portfolio policies, see our Editorial Policies and the Editorial Policy Checklist.

Statistics

For all statistical analyses, confirm that the following items are present in the figure legend, table legend, main text, or Methods section.

n/a	Confirmed	
☐	☒	The exact sample size (n) for each experimental group/condition, given as a discrete number and unit of measurement
☐	☒	A statement on whether measurements were taken from distinct samples or whether the same sample was measured repeatedly
☐	☒	The statistical test(s) used AND whether they are one- or two-sided<br>*Only common tests should be described solely by name; describe more complex techniques in the Methods section.*
☐	☒	A description of all covariates tested
☐	☒	A description of any assumptions or corrections, such as tests of normality and adjustment for multiple comparisons
☐	☒	A full description of the statistical parameters including central tendency (e.g. means) or other basic estimates (e.g. regression coefficient) AND variation (e.g. standard deviation) or associated estimates of uncertainty (e.g. confidence intervals)
☐	☒	For null hypothesis testing, the test statistic (e.g. F, t, r) with confidence intervals, effect sizes, degrees of freedom and P value noted<br>*Give P values as exact values whenever suitable.*
☒	☐	For Bayesian analysis, information on the choice of priors and Markov chain Monte Carlo settings
☒	☐	For hierarchical and complex designs, identification of the appropriate level for tests and full reporting of outcomes
☒	☐	Estimates of effect sizes (e.g. Cohen's d, Pearson's r), indicating how they were calculated

Our web collection on statistics for biologists contains articles on many of the points above.

Software and code

Policy information about availability of computer code

Data collection	The data collection of this study used questionnaires and clinical lab tests. The collected information was imported into Stata/MP (version 17.0) to save as Stata datasets.
Data analysis	R (version 4.2.2) and Stata/MP (version 17.0) was used for all data analyses and presentation of results, with several packages utilized. The 'haven' package (version 2.5.1) was used to import stata files into R, while 'readxl' (version 1.4.1) was used for importing excel files into R. Additionally, the 'openxlsx' package (version 4.2.5.2) was used for writing and styling Excel xlsx files from R, while 'fdrtools' (version 1.2.17) was used to estimate false discovery rates for multiple test correction. Furthermore, 'survivalROC' (version 1.0.3.1) was utilized to create time-dependent ROC curves from censored survival data, using the Nearest Neighbor Estimation (NNE) method of Heagerty, Lumley, and Pepe (2000). For survival analysis in R, the 'survival' package (version 3.4.0) was used, and the 'pROC' package (version 1.18.0) was utilized for visualizing, smoothing, and comparing receiver operating characteristic (ROC) curves. Additionally, data visualisations were created using the 'ggplot2' package (version 3.4.2), with extra themes, scales, and geoms for 'ggplot2' provided by the 'ggthemes' package (version 4.2.4). The 'survminer' package (version 0.4.9) was used for drawing survival curves in 'ggplot2', while 'survIDINRI' (version 1.1.2) was used for comparing competing risk prediction models with censored survival data. To calculate and plot decision curves, the 'ggDCA' package (version 1.1) was utilized, while 'ggprism' (version 1.0.4) provided a 'ggplot2' extension. Finally, the 'patchwork' package (version 1.1.2) was used as a composer of plots. The codes used for the data analyses are available on Github (https://github.com/Yangshp5/RPEMet).<br><br>The association between RPET metabolic fingerprints and the risk of T2DM was evaluated using CPH models, which was adjusted for the same covariates as in the phase-I analysis, with the BH method for multiple testing correction. The RPET metabolic state model is a stepwise CPH model trained on (1) all RPET-associated metabolic biomarkers; (2) only biomarkers that showed negative associations with RPET and positive associations with T2DM risk; and (3) only biomarkers independent of ageing. Participants in the testing set were divided into four quartiles based on the calculated RPET metabolic states, and the risks of developing T2DM were compared among groups. To assess the predictivity of

the RPET metabolic states for T2DM, the Harrell's C-statistics was calculated, and their predictive value for T2DM was compared with those of individual clinical indicators. The added predictability of these metabolites for the risk of T2DM was also evaluated compared to the clinical indicators-based model. The NRIs and IDIs were also computed. The goodness of model fit was assessed using Hosmer-Lemeshow test. Finally, decision curve analyses were conducted to estimate the benefits in clinical utility.

For manuscripts utilizing custom algorithms or software that are central to the research but not yet described in published literature, software must be made available to editors and reviewers. We strongly encourage code deposition in a community repository (e.g. GitHub). See the Nature Portfolio guidelines for submitting code & software for further information.

Data

Policy information about availability of data

All manuscripts must include a data availability statement. This statement should provide the following information, where applicable:

- Accession codes, unique identifiers, or web links for publicly available datasets
- A description of any restrictions on data availability
- For clinical datasets or third party data, please ensure that the statement adheres to our policy

All the data utilized in this study, including imaging, NMR, and genotyping data from the UKB, are available via data access procedures (http://www.ukbiobank.ac.uk). Permission to use the UKB Resource was obtained via a material transfer agreement as part of Application 62443, 62489, 62491 and 62525. Raw data from the GDES analyzed in the current study are not publicly available due to HIPAA compliance and were used with Zhongshan Ophthalmic Center institutional permission for the purposes of this project. All requests for access to in-house data will be addressed to the corresponding authors, Dr. Wei Wang (Email: wangwei@gzzoc.com), and will be processed in accordance with Zhongshan Ophthalmic Center guidelines. Guangzhou Diabetic Eye Study Group will assess all requests based on the purpose of data request, and it may take up to 90 days to process the request. A material-transfer or data-usage agreement will be required between Zhongshan Ophthalmic Center and the receiving organization, and the requesting organization must state the intended purpose of the data transfer and provide assurances that the transferred data will only be used for non-commercial academic and educational purposes in compliance with Zhongshan Ophthalmic Center institutional guidelines. Source data are provided as a Source Data file. Source data supporting all our findings (Figure 2–5 and Supplemental Figure S1–S3) are provided with this publication as a Source Data file. Source data are provided in this paper.

Research involving human participants, their data, or biological material

Policy information about studies with human participants or human data. See also policy information about sex, gender (identity/presentation), and sexual orientation and race, ethnicity and racism.

Reporting on sex and gender	We use the term sex to indicate biological attributes and the term gender to describe the attribute shaped by social and cultural circumstances. Our study included participants of all sexes and genders. There was no exclusion based on sex or gender. The sex information is acquired from central registry at recruitment, but in some cases updated by the participant. Hence it may contain a mixture of the sex the National Health Service (NHS) had recorded for the participant and self-reported sex. Sex-specific analyses has been conducted in our study. Our study included participants of all sexes. Sex-specific analyses were conducted for the associations of in vivo measurements of RPE thickness and incident type 2 diabetes mellitus (Supplementary Table S2). These analyses demonstrated consistent associations across both sexes. Information on gender is not collected. Our analytical results apply to both sexes. To control potential confounding stemming from sex, we have included sex as covariates in all analyses. The distribution of sex was reported in Supplementary Table S1 of the manuscript.
Reporting on race, ethnicity, or other socially relevant groupings	The ethnicity categories used in this study included White, South Asian, East Asian, Black, and Mixed races. The collection of information on ethnicity was critical to the study since previous research has demonstrated that ethnicity differences can confound retinal pigment epithelium (RPE) thickness measurements. The ethnicity categories were determined based on a series of branching questions asked during the initial Assessment Centre visit as part of the touchscreen questionnaire (https://biobank.ctsu.ox.ac.uk/crystal/field.cgi?id=21000). To control for potential confounding variables, this study employed multiple approaches, which are described in detail in the Methods section.
Population characteristics	Baseline characteristics of the study population are summarized in Table 1. Compared to population-II, participants in population-I were typically younger, male, more educated, had higher income, lower BMI, smoked less, and less likely to be hypertension (all $P < 0.05$). Participants in the training and testing sets shared similar distributions of characteristics.
Recruitment	The UKB is a large population-based prospective cohort that recruited approximately 500,000 participants aged 40-73 years in the United Kingdom. Participants visited one of 22 assessment centers across England, Scotland, and Wales. No selection criteria were applied to the NMR metabolomics sampling process. As a result, the participants underwent metabolomic profiling are a random subset of the full UKB cohort.
Ethics oversight	The study was approved by the Northwest Multicenter Research Ethics Committee (11/NW/0382) and the Ethics Committee of Zhongshan Ophthalmic Center (2017KYPJ094), with written informed consent obtained from all participants. The study adheres to the Guidelines of the Ministry of Science and Technology (MOST) for the Review and Approval of Human Genetic Resources.

Note that full information on the approval of the study protocol must also be provided in the manuscript.

Field-specific reporting

Please select the one below that is the best fit for your research. If you are not sure, read the appropriate sections before making your selection.

☒ Life sciences ☐ Behavioural & social sciences ☐ Ecological, evolutionary & environmental sciences

For a reference copy of the document with all sections, see nature.com/documents/nr-reporting-summary-flat.pdf

Life sciences study design

All studies must disclose on these points even when the disclosure is negative.

Sample size	This is a cohort study that assesses the exposure-outcome associations. In this case, the sample size is determined by the number of events required to perform multivariable regression models. According to the rule-of-thumb estimation, at least ten events are required per variable (including dummy variables) in the model (Riley et al. BMJ. 2020). In the fully adjusted models, we included a total of 30 variables (including dummy variables) in the Cox regression models. Thus, at least 330 events for each outcome were required. The events for type 2 diabetes were 4,038 in the training set and 1,676 in the testing set, respectively. Therefore, the sample size of this study should be sufficient.
Data exclusions	The eligibility criteria involved the exclusion of participants without NMR metabolomic profiling and OCT scanning , as well as participants with missing thickness values, low signal strength, poor centration or segmentation, high refractive error (spherical equivalent [SE] >6 or <-6 diopters [D]), visual impairment (> 0.1 logarithm of the minimum angle of resolution [log MAR]), or abnormal intraocular pressure (IOP) (≥ 22 or ≤ 5 mmHg). Furthermore, patients with glaucoma, other retinal disorders (including retinal detachments and breaks, retinal vascular occlusions, and others), and neurodegenerative diseases were excluded, owing to the potential for secondary retinal destruction.
Replication	This is a population-based epidemiological cohort study, the findings of which may need to be validated by further clinical trials and/or larger cohorts.
Randomization	As an observational cohort study, randomization is not applicable to this research. However, to minimize potential confounding, a variety of covariates were carefully taken into account, simulating randomization as closely as possible.
Blinding	This is a population-based epidemiological cohort study which does not perform blinding to give treatments or placebo as randomized controlled trials

Reporting for specific materials, systems and methods

We require information from authors about some types of materials, experimental systems and methods used in many studies. Here, indicate whether each material, system or method listed is relevant to your study. If you are not sure if a list item applies to your research, read the appropriate section before selecting a response.

Materials & experimental systems

n/a	Involved in the study	
☒	☐	Antibodies
☒	☐	Eukaryotic cell lines
☒	☐	Palaeontology and archaeology
☒	☐	Animals and other organisms
☒	☐	Clinical data
☒	☐	Dual use research of concern
☒	☐	Plants

Methods

n/a	Involved in the study	
☒	☐	ChIP-seq
☒	☐	Flow cytometry
☒	☐	MRI-based neuroimaging
